# The size-distribution of Earth’s lakes

**DOI:** 10.1038/srep29633

**Published:** 2016-07-08

**Authors:** B. B. Cael, D. A. Seekell

**Affiliations:** 1 Massachusetts Institute of Technology, Cambridge, MA 02139, USA; 2 Woods Hole Oceanographic Institution, Woods Hole, MA 02543, USA; 3 Umeå University, 901 87 Umeå, Sweden

## Abstract

Globally, there are millions of small lakes, but a small number of large lakes. Most key ecosystem patterns and processes scale with lake size, thus this asymmetry between area and abundance is a fundamental constraint on broad-scale patterns in lake ecology. Nonetheless, descriptions of lake size-distributions are scarce and empirical distributions are rarely evaluated relative to theoretical predictions. Here we develop expectations for Earth’s lake area-distribution based on percolation theory and evaluate these expectations with data from a global lake census. Lake surface areas ≥8.5 km^2^ are power-law distributed with a tail exponent (*τ* = 1.97) and fractal dimension (*d* = 1.38), similar to theoretical expectations (*τ* = 2.05; *d* = 4/3). Lakes <8.5 km^2^ are not power-law distributed. An independently developed regional lake census exhibits a similar transition and consistency with theoretical predictions. Small lakes deviate from the power-law distribution because smaller lakes are more susceptible to dynamical change and topographic behavior at sub-kilometer scales is not self-similar. Our results provide a robust characterization and theoretical explanation for the lake size-abundance relationship, and form a fundamental basis for understanding and predicting patterns in lake ecology at broad scales.

Visual examination of maps or satellite images reveals the size-distribution of Earth’s lakes is strongly skewed: there are many small lakes, but few large lakes [refs 1, 2, 3, 4, 5; Fig. 1]. Most key ecosystem processes in lakes scale strongly with lake surface area, and therefore, the size-distribution of lakes is a key constraint on patterns in lake ecology and biogeochemistry^3^,^4^,^5^,^6^. In particular, an accurate characterization of Earth’s lake size-distribution is critical for estimating global rates of lake productivity and greenhouse gas emissions^2^,^3^,^4^,^5^,^6^,^7^,^8^,^9^,^10^. Most variation in estimates of these global rates is related to changes in estimates of the abundance and size-distribution of lakes, not revisions in the areal rates of biological and biogeochemical processes themselves^7^. Despite this fundamental interest and practical importance, there remain few rigorous evaluations of Earth’s lake size-distribution^1^,^4^,^5^.

It is widely believed that Earth’s lake areas are power-law distributed, but evidence supporting this belief has been inconsistent^1^,^2^,^3^,^4^,^5^. On one hand, lake shorelines are fractal. Fractality and power-law distributions are inextricably linked because both are a result of scale invariance^10^,^11^,^12^. Additionally, linear regressions on log-abundance log-area plots achieve extremely high levels of explained variance, which is consistent with a power-law size-distribution^1^,^3^,^4^,^5^,^10^. On the other hand, high levels of explained variance on log-abundance log-area plots have been identified as unreliable criteria for identifying power-laws and few studies apply formal statistical tests for power-law distributions^11^,^13^,^14^. Further, simulation studies do not produce all of the patterns expected from fractal predictions, and empirical distributions are rarely compared to theoretical expectations^1^,^11^,^15^,^16^. Collectively, these inconsistencies indicate that Earth’s lake-size distribution is inadequately characterized both empirically and theoretically. Here, we characterize this distribution by developing expectations based in percolation theory. We then test these expectations using lake size data from a global lake census developed from high-resolution satellite imagery.

Percolation theory is a canonical branch of statistical physics that provides a plausible idealized description of lake geometry and the processes contributing to the asymmetry in the lake size-abundance relationship^17^,^18^,^19^. In these models, ‘filled’ area that represents standing water is randomly distributed over a domain. This can be modeled as distribution over random sites in a discrete lattice, into randomly located overlapping circles in a plane, or into local minima of a surface with random heights. Connected or overlapping filled regions are called percolation clusters, which are a type of random fractal^18^. Clusters are statistically self-similar over certain ranges of area dependent on the minimum site size and the proportion of filled area [ref. 18; Supplementary Information]. Over these ranges, the cluster areas take a power-law size distribution with a predictable exponent that is universal across percolation problems [refs 18, 19, 20, 21]. A heuristic explanation for this is given in the Supplementary Information. Geological random fractals are not power-law distributed across their full range of areas because of physical constraints; for instance, the finite extent of Earth’s surface constrains the maximum possible lake size^11^,^12^,^16^. For small lakes, deviation from a power-law distribution could occur if scale dependent geomorphic processes like mass wasting override general topographic characteristics^11^,^16^,^22^,^23^,^24^. Measuring lake statistics - whether lakes conform to a power law distribution, over what range, with what exponent, and what fractal behavior - is the principal approach for connecting empirical observations to theory^16^,^17^,^25^.

The theoretical critical power-law distribution tail exponent for percolation cluster sizes is *τ* = 187/91, or approximately 2.05 refs 18,25. The boundaries, or ‘unscreened perimeters’, of the percolation clusters are analogous to lake shorelines^26^. Conceptually, the unscreened perimeter indicates that two lakes connected by a stream would be considered independent features with separate surface areas and perimeters^20^. This description is important because it allows predictions from percolation theory to account for both lakes connected to riverine hydrologic networks and those that are disconnected because they lack inlets and outlets. Most theoretical analyses of lake size-distributions assume that rivers connect all lakes^12^,^15^. This is not an accurate assumption for Earth’s lakes and hence predictions based on the unscreened perimeter of percolation clusters are advantageous. Another advantage of considering unscreened perimeters is that they are fractals, creating an additional testable expectation. Specifically, the fractal scaling behavior of lake perimeters (*l*) is based on their surface area (*a*): 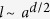. Based on this relationship, fractal dimension *d* for unscreened perimeters is expected to be *d* = 4/3 ref. 25. Hence, percolation theory provides three testable hypotheses about Earth’s lake size-distribution: 1) lake areas are power-law distributed over a portion of the total range of lake areas, 2) for this portion the lake size distribution has a tail exponent *τ* = 2.05, and 3) for this portion the fractal dimension of shorelines based on perimeter-area dimensional analysis is *d* = 4/3.

Our analysis finds Earth’s lakes are power-law distributed for lakes ≥8.5 km^2^ (Fig. 2). Lakes <8.5 km^2^ deviate from the power-law distribution such that there are fewer of these small lakes than expected if small lakes conformed to the same power-law distribution as large lakes. Across the range of lake sizes conforming to a power-law distribution, the tail exponent is *τ* = 1.97 (+/−0.01), close to the predicted value *τ* = 2.05. The shorelines of these lakes have a fractal dimension *d* = 1.38 close to the predicted value *d* = 4/3 (Fig. 3). The deviation from power-law form begins two orders of magnitude larger then the minimum reliably mapped lake size, a strong indicator that the observed deviation is not due to sampling biases^13^,^27^. This deviation is consistent with a conceptual representation of lakes as geologic random fractals, which are not power-law distributed across their full range of sizes^18^.

We also examined the size distribution of lakes in the Swedish national lake registry, which is considered a complete census of lakes in Sweden^27^,^28^. A key benefit of examining this dataset is that it is based on map compilations that integrate different types of errors compared to the global lake census which was developed based on an automated algorithm applied to remotely sensed data^2^. The Swedish lake data were power-law distributed for lakes larger, but not smaller, than 4.7 km^2^ (Fig. 2). Similar to the global lake data, this deviation begins several orders of magnitude above the minimum reliably mapped lake size. The tail exponent over the power-law range was 2.13 (+/−0.04), similar to theoretical predictions (2.05) and empirical results from the global data (1.97). Shorelines of lakes ≥4.7 km^2^ have a fractal dimension *d* = 1.34 very similar to the predicted values of *d* = 4/3 (Fig. 3); lakes <4.7 km^2^ have a fractal dimension *d* = 1.00. Hence, these results support our characterization of Earth’s lake size-distribution and minimize lake census methodology as a potential factor influencing this characterization.

Collectively, our results characterize Earth’s lake size-distribution and demonstrate consistency with predictions based on percolation theory. There are two distinct modes of lake behavior; above the order of 1 km^2^ for which lakes behave as power-law distributed fractals similar to a percolation process, while below the order of 1 km^2^ for which lake abundance does not conform to a power-law. This deviation has not previously been characterized, but is an important constraint on the relative contributions of small versus large lakes to the total surface area of lakes. Specifically, the power-law tail exponents predicted by percolation theory and measured here empirically are consistent with small lakes dominating the total surface area of Earth’s lakes^1^. However, the deviation from power-law for small lakes precludes this and creates a pattern whereby medium sized lakes dominate the total lake surface area^1^,^2^. Geographic patterns of area-dependent ecosystems rates and characteristics are therefore constrained by both the tail exponent and transition scale characterized by our study^4^,^5^.

The deviation of small lakes from a power-law distribution is of great practical importance^11^,^13^. Estimates global lake productivity and green house gas emissions are generally estimated in part by calibrating a power-law distribution based on large lake areas, and then using this distribution to extrapolate the size and abundance of small lakes^7^,^8^,^9^,^10^,^11^,^13^. If lakes conformed to a power-law distribution across the full range of lake sizes, there would be an order of magnitude more lakes sized 0.01–1 km^2^ than exists on Earth’s surface. The reason for this deviation is likely in part because Earth’s topography exhibits autocorrelation at small scales^24^. An average value for this transition is 0.9 km, which is similar to the transition scale in our lake analysis [ref. 24 and references therein]. Other scale-dependent factors probably also contribute to the under-abundance of small lakes relative to a power-law distribution. For example small lakes fill more rapidly than large lakes, and this disproportionately rapid filling is enhanced by human activity^3^,^23^. New remote sensing approaches are reducing uncertainty in the abundance and size-distribution of small lakes but a strong conceptual basis is still necessary because observed distributions of small lakes do not conform to most common statistical distributions^2^,^11^,^13^. A potentially fruitful area of research may be in the interactions between geomorphological processes filling in lakes and the distribution of lake ages^22^. Highly skewed age distributions might exist for lakes because most are glacial and young, but some are tectonic and old. Theoretical analyses suggest, in a general sense, skewed age distributions can result in observed size-distributions that strongly contrast those predicted by process-based models^29^. This contrast is especially strong for small sizes^29^.

The generality of percolation theory as a minimal model may explain why many of the characteristics of lakes on other planetary bodies have similarities with Earth’s lake size-distribution^18^,^30^. For example, ancient lake basins on Mars are power-law distributed over some, but not all, ranges of surface area^11^,^13^. These lakes have fractal dimensions similar to values expected based on Mars surface topography^11^. On Titan, hydrocarbon lakes have similar morphological characteristics to Earth’s lakes^30^. While limited imagery precludes a detailed evaluation of Titan’s lake size-distribution, fractal dimensions for individual lake shorelines are very similar to those predicted by percolation theory and observed for Earth’s lakes^30^. These observations suggest that the processes controlling the statistical aspects of lake geometry are not limited to the current biological, hydrological, and geological conditions on Earth.

In conclusion, the asymmetry between area and abundance is a fundamental constraint on broad-scale patterns in lake ecology. Our results provide a robust characterization and theoretical explanation for the asymmetry in lake size-abundance relationship. These results provide the fundamental basis necessary for understanding and predicting patterns in lake ecology at broad scales.

## Methods

We analyzed lake areas and perimeters from a global and a regional lake census^2^,^28^. The two databases were developed independently, although the regional lake census has served as a validation case for the global census^27^. The global lake census, described in detail in ref. 2, is based on an automated lake extraction algorithm applied to high-resolution multispectral satellite imagery. The automated algorithm has been shown to be highly accurate including for estimates of lake surface area and perimeter. The global lake census does not include the Caspian Sea and therefore it is also excluded from our analysis. The regional lake census is based on compilations of maps and is considered a complete record of Swedish lakes ≥0.01 km^2^ ref. 28.

For the power-law distribution tests, we applied established statistical methods described in detail in ref. 16. These methods are advantageous over those typically used to evaluate lake size distributions. Specifically, lake size distributions are typically evaluated using ordinary least squares regression on log-abundance log-size data, which is useful for demonstrating a size-abundance asymmetry, but is known to mischaracterize the lake size distribution by exponentially overweighting the influence of small features, unless deviations are lognormal distributed^14^. Furthermore, ordinary least squares provides no objective method for estimating the minimum value above which data are power-law distributed, thus a low cutoff is typically not tested for in analyses of lake size distributions^1^,^5^,^13^,^16^. The approach that we applied not adversely impacted by weighting and provides accurate tail exponent measurements; additionally, this approach estimates a minimum value for the estimated distribution^14^, which is on the order of 1 km^2^ for both data sets [Supplementary Information].

Shoreline fractal dimensions were computed based on dimensional analysis, comparing perimeter (*l*) with the square root of area (*a*^*1/2*^). For Swedish lakes, as deviations exist in both perimeter and area measurements and are not normally distributed, a robust statistics methodology is required; we applied bi-square regression on a nonlinear least squares power-law fit. For global lakes ≥8.5 km^2^, however, an ordinary least squares regression of log(*l*) vs. log(*a*^*1/2*^) was found to have normal deviations (kurtosis = −0.037, skewness = −0.32, Kolmogorov-Smirnov test = 0.036), thus ordinary least squares regression is the maximum likelihood estimator for these data and robust statistics were not required. Global lakes <8.5 km^2^ are too spread to have a well-defined fractal dimension; no fit explains a satisfactory portion of variance.

## Additional Information

**How to cite this article**: Cael, B. B. and Seekell, D. A. The size-distribution of Earth’s lakes. *Sci. Rep*. **6**, 29633; doi: 10.1038/srep29633 (2016).

## Supplementary Material

Supplementary Information

## Figures and Tables

**Figure 1:**
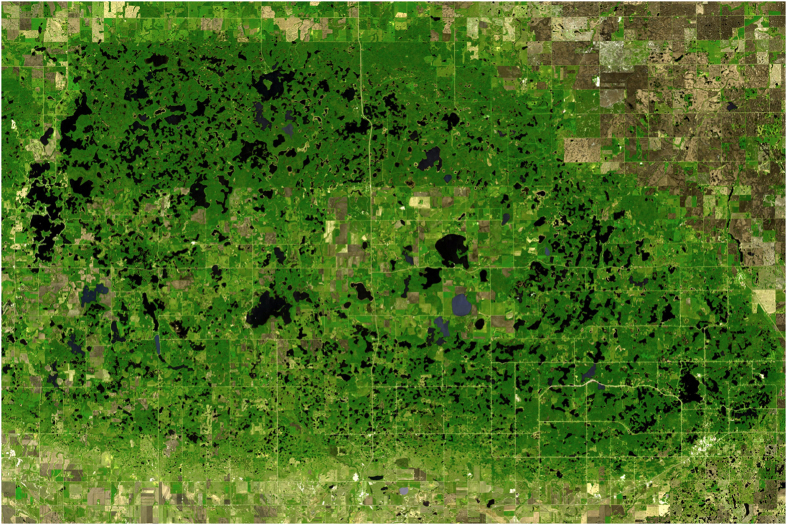
Globally, there are many small lakes, but only a small number of large lakes. The asymmetry between lake size and abundance is clearly visible in this image of the Turtle Mountains, a lake district in North Dakota (USA). Image courtesy NASA.

**Figure 2:**
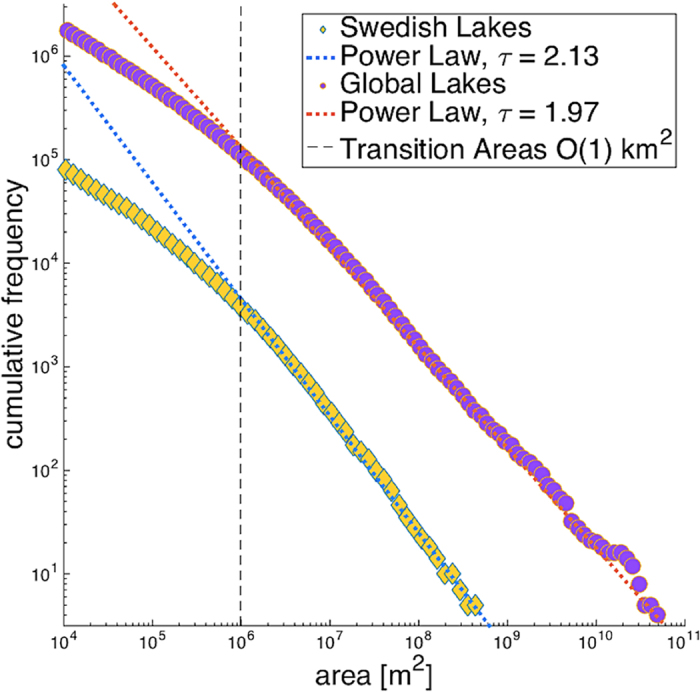
Log-abundance (cumulative frequency) log-area plots of global and Swedish lakes illustrate the asymmetry between lake size and abundance. Large lakes are power-law distributed and the tail exponents for both datasets are very similar to the *τ* = 2.05 value predicted by percolation theory. Both lake size-distributions deviate from a power-law for lakes with surface areas smaller than 1 km^2^. This deviation is to the extent that there are an order of magnitude fewer lakes 0.01–1 km^2^ recorded in these datasets than would be expected if lakes conformed to a power-law size distribution across their full range of sizes.

**Figure 3:**
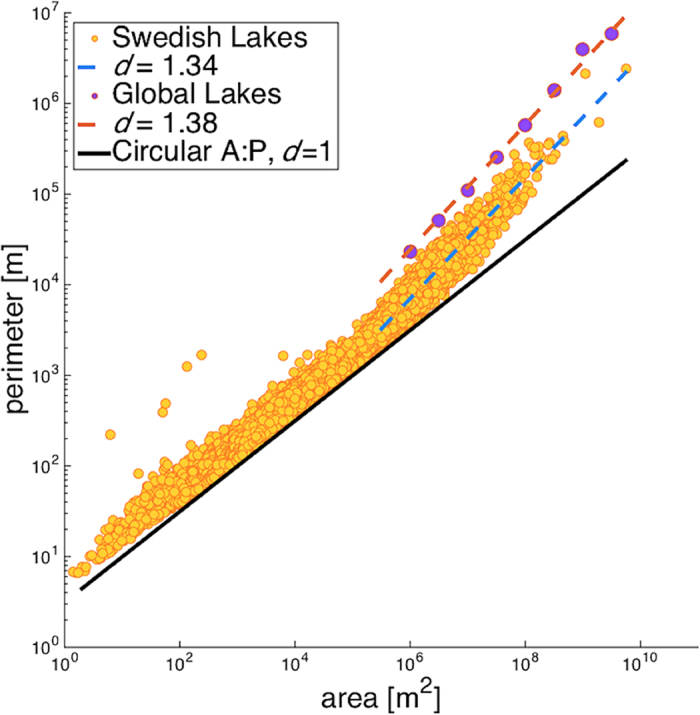
Fractal dimensions derived from perimeter-area relationships. The black line is a physical constraint where shorelines are circular and have a fractal dimension *d* = 1. Lakes cannot fall below the black line. For context, comparing lakes falling across the black line is equivalent to comparing circles with different areas. Comparing lakes across the observed perimeter-area relationships reveals that more convoluted shorelines are observed for larger lakes. In this figure, Swedish lake data are plotted to emphasize the variation and transition in values. Global lake data are plotted as logarithmically binned median values because the size of the database precludes plotting individual records.
