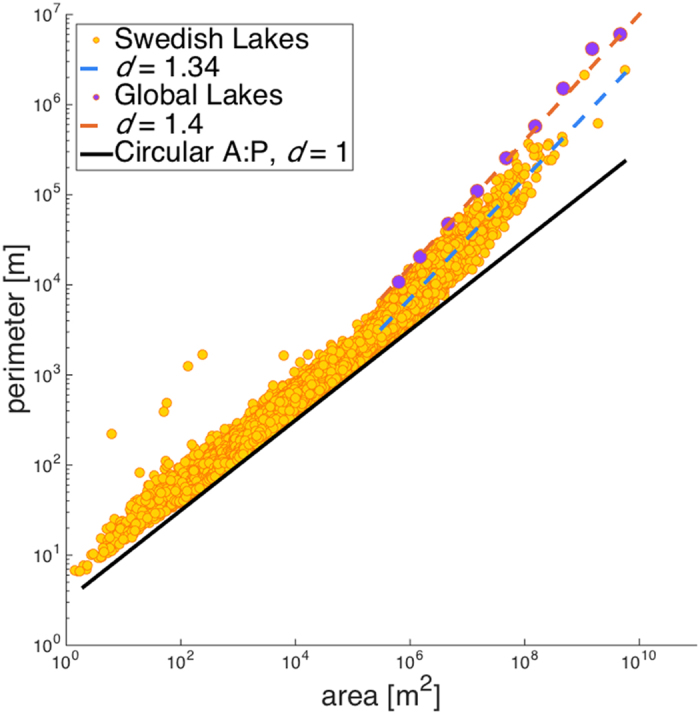# Corrigendum: The size-distribution of Earth’s lakes

**DOI:** 10.1038/srep42155

**Published:** 2017-02-13

**Authors:** B. B. Cael, D. A. Seekell

Scientific Reports 6: Article number: 2963310.1038/srep29633; published online: 07 08 2016; updated: 02 13 2017

The original version of this Article contained errors. A subset of the global lake census was incorrectly and inadvertently used instead of the full dataset resulting in three parameters to be incorrect: power-law slope for the lake size-distribution, the area at which small lakes begin to deviate from a power-law distribution, and the fractal dimension relating lake areas to perimeters.

In the Abstract,

“Lake surface areas ≥8.5 km^2^ are power-law distributed with a tail exponent (*τ* = 1.97) and fractal dimension (*d* = 1.38), similar to theoretical expectations (*τ* = 2.05; *d* = 4/3). Lakes <8.5 km^2^ are not power-law distributed”.

now reads:

“Lake surface areas ≥0.46 km^2^ are power-law distributed with a tail exponent (*τ* = 2.14) and fractal dimension (*d* = 1.4), similar to theoretical expectations (*τ* = 2.05; *d* = 4/3). Lakes <0.46 km^2^ are not power-law distributed”.

In the Introduction section,

“Our analysis finds Earth’s lakes are power-law distributed for lakes ≥8.5 km^2^ (Fig. 2). Lakes <8.5 km^2^ deviate from the power-law distribution such that there are fewer of these small lakes than expected if small lakes conformed to the same power-law distribution as large lakes. Across the range of lake sizes conforming to a power-law distribution, the tail exponent is *τ* = 1.97 (+/−0.01), close to the predicted value *τ* = 2.05. The shorelines of these lakes have a fractal dimension *d* = 1.38 close to the predicted value *d* = 4/3 (Fig. 3)”.

now reads:

“Our analysis finds Earth’s lakes are power-law distributed for lakes ≥ 0.46 km^2^ (Fig. 2). Lakes < 0.46 km^2^ deviate from the power-law distribution such that there are fewer of these small lakes than expected if small lakes conformed to the same power-law distribution as large lakes. Across the range of lake sizes conforming to a power-law distribution, the tail exponent is *τ* = 2.14 close to the predicted value *τ* = 2.05. The shorelines of these lakes have a fractal dimension *d* = 1.4 close to the predicted value *d* = 4/3 (Fig. 3)”.

Figure 2 is incorrect. The y-axis is incorrect for global data as a subset of total lakes was inadvertently used for analysis. The Power Law *τ* = 2.14 was incorrectly given as *τ* = 1.97. The correct Figure 2 appears below as Figure 1. The legend of Figure 2 is incorrect,

“Log-abundance (cumulative frequency) log-area plots of global and Swedish lakes illustrate the asymmetry between lake size and abundance. Large lakes are power-law distributed and the tail exponents for both datasets are very similar to the *τ* = 2.05 value predicted by percolation theory. Both lake size-distributions deviate from a power-law for lakes with surface areas smaller than 1 km^2^. This deviation is to the extent that there are an order of magnitude fewer lakes 0.01–1 km^2^ recorded in these datasets than would be expected if lakes conformed to a power-law size distribution across their full range of sizes”.

now reads:

“Abundance (cumulative frequency) - area plots of global and Swedish lakes illustrate the asymmetry between lake size and abundance. Large lakes are power-law distributed and the tail exponents for both datasets (*τ* = 2.13, *τ* = 2.14) are very similar to the *τ* = 2.05 predicted by percolation theory. Both lake size-distributions deviate from a power-law for lakes with surface areas smaller than ~1 km^2^. This deviation is to the extent that there are more than a factor of three fewer lakes 0.01–1 km^2^ recorded in these datasets than would be expected if lakes conformed to a power-law size distribution across their full ranges. Lake abundance of Swedish lakes has been rescaled by a factor of 100 in the above image so as to coincide with the cumulative distribution for global lake abundance”.

Figure 3 is incorrect. The fractal dimension *d* = 1.4 is incorrectly given as *d* = 1.38. The correct Figure 3 appears below as Figure 2. The Figure legend is correct.

In addition, in the Introduction section,

“The tail exponent over the power-law range was 2.13 (+/−0.04), similar to theoretical predictions (2.05) and empirical results from the global data (1.97)”.

now reads:

“The tail exponent over the power-law range was 2.13 (+/−0.04), similar to theoretical predictions (2.05) and empirical results from the global data (2.14)”.

In the Methods section,

“For Swedish lakes, as deviations exist in both perimeter and area measurements and are not normally distributed, a robust statistics methodology is required; we applied bi-square regression on a nonlinear least squares power-law fit. For global lakes ≥8.5 km^2^, however, an ordinary least squares regression of log(*l*) vs. log(*a*^*1/2*^) was found to have normal deviations (kurtosis = −0.037, skewness = −0.32, Kolmogorov-Smirnov test = 0.036), thus ordinary least squares regression is the maximum likelihood estimator for these data and robust statistics were not required. Global lakes <8.5 km^2^ are too spread to have a well-defined fractal dimension; no fit explains a satisfactory portion of variance”.

now reads:

“For both Swedish and global lakes, as variability exists in both perimeter and area measurements and residuals are not normally distributed, a robust statistics methodology is required. We applied robust regression with each of the standard weighting functions available in MATLAB (version R2014b). For global lakes, the estimate of *d* was sensitive both to weighting functions and to factor-of-two changes in the cut off threshold of 0.46 km^2^, varying between 1.39 to 1.44; we thus report a more conservative estimate of *d* = 1.4 for global lakes above this threshold. Swedish lakes’ *d* estimate exhibited neither such sensitivity; we thus report an estimate of *d* = 1.34 for Swedish lakes >4.7 km^2^. Swedish lakes <4.7 km^2^ have a fractal dimension of *d* = 1.00 according to the same procedure. Global lakes <0.46 km^2^ are too spread to have a well-defined fractal dimension; no fit explains a satisfactory portion of variance”.

These errors have now been corrected in the PDF and HTML versions of the Article.[Fig f1][Fig f2]

## Figures and Tables

**Figure 1 f1:**
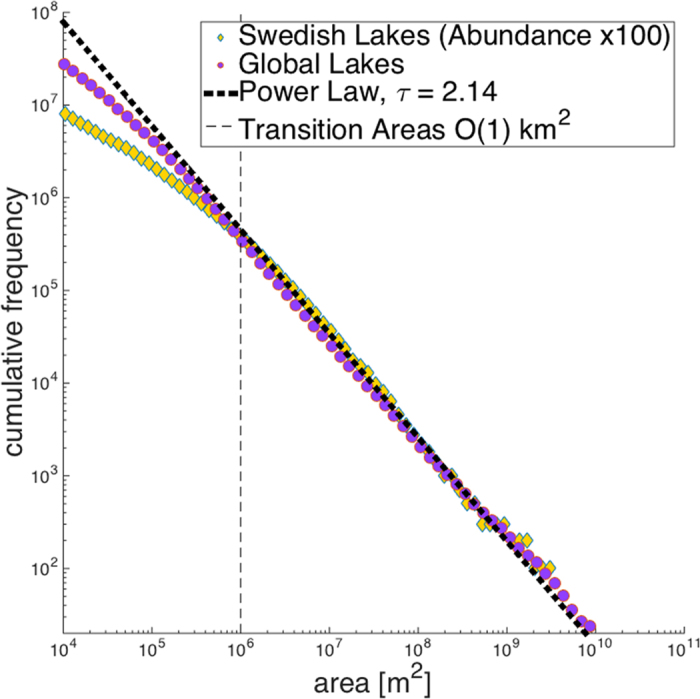


**Figure 2 f2:**