# Supplementary material for: The size-distribution of Earth’s lakes
Source: Sci Rep. 2016 Jul 8;6:29633. doi: 10.1038/srep29633 (PMC4937396; doi:10.1038/srep29633)
Supplement: Supplementary Information [file srep29633-s1.doc]

**The size-distribution of Earth’s lakes**

B. B. Cael^1,2,*^ and D. A. Seekell^3^

1. Massachusetts Institute of Technology, Cambridge, MA 02139 USA
2. Woods Hole Oceanographic Institution, Woods Hole, MA 02543 USA
3. Umeå University, 901 87 Umeå, Sweden

* Corresponding author, email: bcaelb@mit.edu

**Supplementary Information**

In this Supplemental text, we give a brief heuristic explanation of how percolation theory results in a power law distribution of percolation clusters, and why this model might apply to lakes scales at which topography is self-similar. Additionally, we discuss why our predicted and observed power-law tail exponents are different from those in previous studies.

Why are percolation clusters power-law distributed?

Consider the case of a 2D grid of square cells, each with a probability *p* of being filled*.* There is critical probability *p_c_* at which an infinite cluster appears (i.e. a connected grouping of filled cells stretches between boundaries of the 2D grid). If *p* is smaller than the critical probability *p_c_*, there is a finite size *G* above which clusters are ‘exponentially scarce’ [21] (Here this quote from ref. 21 is relevant: ‘The correlation length [*G^1/2^*] is also the upper bound of the scaling range where percolation clusters behave self-similarly and hence may be characterized by a fractal dimension.’). Additionally, clusters of connected, filled cells cannot be smaller than a single grid cell area *g*. We can then expect that clusters with area close to *g* will be sensitive to the specific details of the lattice, e.g. the probability of finding a cluster of area *g* is straightforwardly *p(1-p)^4^* in this grid because it requires finding a filled grid cell with unfilled adjacent cells. Similarly, clusters with area close to *G* are pushing up against non-occurrence. In between these extremes, however, we can rescale the lattice as follows, and it will preserve its characteristics, indicating self-similarity in this range.

Consider a large lattice of size *L* x *L*, where each cell is filled with probability *p < p_c_*. Now replace each 2 x 2 quartet of sites with a 'supersite' of area 4*g*, which is

1. filled if more than two of the sites comprising it are filled,
2. unfilled if fewer than two sites comprising it are filled, and
3. filled with probability 1/2 if two of the sites comprising it are filled.

Now consider the lattice *L* x *L* as being one quadrant of a larger 2*L* x 2*L* lattice. Statistically, there is no difference between the original lattice and this rescaled 'superlattice', besides that the minimum cluster size is now 4*g*! Thus after rescaling we expect the lattices to behave the same; we expect the supersites to have the same statistical properties as the original sites, besides that the difference between *G* and the minimum cluster size is smaller. The rescaling can also be done in reverse: in this range *g* << cluster size << *G*, clusters are self-similar, and their properties are invariant of scale.


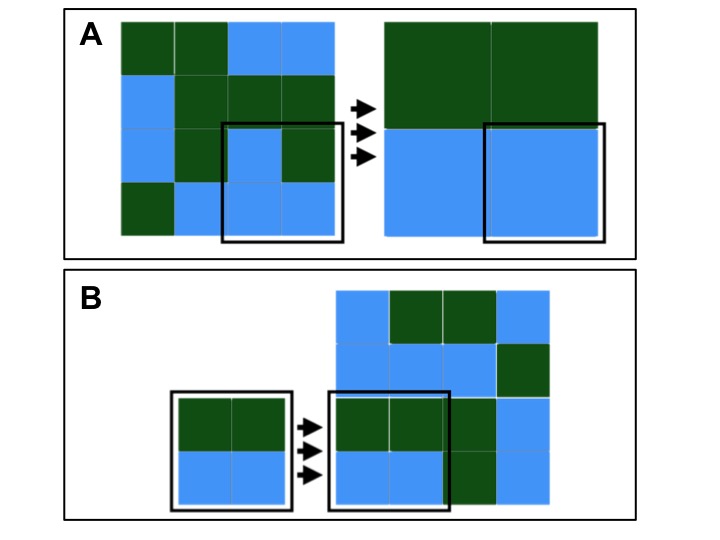


Figure S1. Cartoon illustration of rescaling a hypothetical grid, following rules 1-3 above.

This scale invariance is what produces the power law distribution because a power law is the only distribution which is identical regardless of scale of examination; given a distribution *p(x)* for some quantity *x,* if it satisfies *p(kx) = f(k)p(x)* for any *k>0,* it necessarily is a power law [31]. The exponent of the power law is given by *p(1)/p’(1)*, where the prime denotes a derivative. In Figure S1 we see the rescaled superlattice has the same ratio of 4-clusters to 2-clusters as the original lattice, i.e. *p(16g)/p(8g) = p(4g)/p(2g)* which in general results in *p* being power-law distributed. This illustration is of course not a proof – it is merely indicative of why we might expect such behavior in a certain scale range. Fractals are objects that exhibit the same properties upon rescaling; thus fractal behavior is inextricably linked to power law distributions.

The above example may seem artificial in connection to lakes; lakes are not collections of squares. However, percolation theory is a classical example of a ‘universality class’ in statistical physics, wherein the values for the tail exponent, fractal dimension, and other quantities ‘withstand moderate violence’ such as changing the lattice structure or removing a lattice altogether and working in a continuum setting: ‘in the long run, this universality enables the success in applying percolation theory to a number of problems that seem to be very far from the origin one’ [21]. Models of continuum percolation theory based on randomly placing circles on a plane, or filling a smooth random surface up to a particular level, yield the same behavior because the problems can be mapped from a lattice and back. For instance in the latter case, called the *potential model*, the local minima of the random surface are equivalent to sites on a lattice, and are connected if the ridge between them stays lower than the given filling level [21]. Thus the power-law and fractal behavior are very general, independent of model details, and only require self-similar, random filling.

However, for lakes we may only expect such distributions and fractal behavior where the landscape in which they are embedded allows them to be self-similar. A host of physical processes, e.g. erosion and sediment transport, can limit at smaller scales the self-similarity of topographic features, and can also smooth coastlines, but at larger scales Earth’s topography is often described as self-similar. Transitions to self-similar topographic behavior occur at different scales in different regions – regional heterogeneity is substantial, as would be expected – but typically occur around ~1km scales [10, 32, and refs. therein]. Our lake analysis [Methods] found minimum values for power-law distributions to begin on this order for both Swedish and global lakes, with the Swedish lake power law beginning at 4.7 km^2^ (corresponding to a topographic transition to self-similarity of 2.2 km) and the global lake power law beginning at 8.5 km^2^ (corresponding to a topographic transition to self-similarity of 2.9 km). Thus these transition scales are similar for lakes and the topography in which they are embedded. The tailing of both empirical distributions below the order 1 km^2^ suggests that power-law distributions for lakes without such cutoffs would over predict the abundance of lakes with surface area 0.01-1 km^2^ by an order of magnitude [Figure 2].

Why is the expected tail exponent different from pervious studies?

The expected tail exponent based on percolation theory is *τ* = 187/91. This tail exponent is fit based on the probability density function (pdf). Most previous analyses of lake size-distributions evaluate tail exponents based on the complementary cumulative distribution function (cdf), and these values are 1 less than the slope based on the pdf approach [1, 6, 7, 13, 16, 33, 34]. Hence, our predicted tail exponent is *τ* = 96/91 when adjusted to facilitate comparison with these previous analyses. This tail exponent is outside of the range of plausible values predicted by other analyses [7, 16, 33]. Specifically, previous theoretical tests have made restrictive assumptions about the power spectral density of topography whereby the topography is a fractional Brownian motion [16]. Using this approach, the fractal dimension of shorelines is directly related to the tail exponent of the power-law size-distribution based on the cdf (tail exponent = *d*/2), and therefore the slope of the abundance-size relationship is constrained by the ranges of possible fractal dimensions for lakes shorelines (1≤ *d* <2). The tail exponent predicted based on percolation theory is slightly higher than the upper limit of the range of exponents predicted by statistical topography. While some landscapes meet the assumptions of the statistical topography approach [7], many landscapes do not and have tail exponents outside of range predicted under its assumptions [1, 7, 19, 33]. Our empirical analysis, which integrates regional heterogeneities, demonstrates remarkable consistency with the more general assumptions of percolation theory, but is inconsistent with the predictions from previous studies based on statistical topography [7, 19, 33]. This is illustrative of the advantageous characteristics of the percolation theory approach, which include applicability on periodic surfaces [21]. Such surfaces exist on Earth but violate assumptions of the statistical topography approach [10].

Supplementary References

1. Newman, M. E. J. Power laws, Pareto distributions and Zipf’s law. *Contemp. Phys.* **46**, 323-351 (2005).
2. Dodds, P. S. , & Rothman, D. H. Scaling, universality, and geomorphology. *Ann. Rev. Earth and Planetary Sci.* **28**, 571-610 (2000).
3. Hamilton, S. K., et al. in *Lowland floodplain rivers: Geomorphological perspectives* (eds Carling P. A. & Petts G. E.) 145-163 (Wiley, 1992).
4. Vidondo, B., et al. Some aspects of the analysis of size spectra in aquatic ecology. *Limnol. Oceanogr.* **42**, 184-192 (1997).
